# Risk factors for gut colonization with vancomycin-resistant enterococci among Bulgarian critically ill patients

**DOI:** 10.1186/s13099-023-00564-x

**Published:** 2023-07-26

**Authors:** Preslava M. Hristova, Teodora V. Marinova-Bulgaranova, Tanya V. Strateva, Stefan V. Trifonov, Hristina Y. Hitkova

**Affiliations:** 1 Department of Microbiology and Virology, Medical University - Pleven, Pleven, Bulgaria; 2 Department of Medical Microbiology, Medical University of Sofia, Sofia, Bulgaria; 3 Department of Anatomy, Histology, Cytology and Biology, Medical University - Pleven, Pleven, Bulgaria

**Keywords:** Vancomycin-Resistant Enterococci, Intensive care units, Risk factors

## Abstract

Vancomycin-resistant enterococci (VRЕ) are recognized as important hospital pathogens which have become common in patients admitted to the intensive care units (ICUs). The purpose of this study was to evaluate the incidence of and the risk factors for colonization with VRE among ICU patients. A total of 91 patients who had duration of hospitalization more than 48 h and without infection caused by VRE or/and other microorganisms in the ICU at University Hospital, Pleven were screened for colonization with VRE. The following data were collected: demographic characteristics, clinical information and antimicrobials use. The statistical analysis was performed using SPSS version 27.0. Colonization with VRE was established in 22 patients and one was carrying two enterococcal species. A total of 23 VRE were isolated. The univariate analysis showed that the postoperative critical cares (*p* < 0.001), cardiovascular diseases (*p* = 0.009) and the presence of an endotracheal tube (*p* = 0.003) were risk factors for colonization with VRE. Also, the postoperative critical cares (*p* = 0.021) and cardiovascular diseases (*p* = 0.018) were confirmed as independent risk factor for VRE acquisition by multivariate analysis. The prevalence of VRE colonization among the ICU patients was relatively high (24.2%). Risk factors for acquisition of intestinal VRE were the postoperative cares, cardiovascular diseases and the presence of an endotracheal tube.

## Main text

Vancomycin-resistant enterococci (VRE) are one of the multidrug-resistant pathogens with the highest hospital prevalence. Their ecological plasticity, ability to colonize the human intestinal tract, as well as prolonged decolonization thereafter are prerequisites for their widespread nosocomial dissemination [1]. The colonization with VRE precedes and plays a key role in the epidemiology of enterococcal healthcare-associated infections [2, 3]. The incidence of VRE colonization and infection is highest among patients with bone marrow transplantation and those who are treated in intensive care units (ICUs), while it is insignificant in immunocompetent individuals [4].

The ability of VRE to colonize or/and to infect the human has been increased by the presence of different virulence determinants. Our study focused on the prevalence of genes encoding aminoglycoside resistance and virulence factors among intestinal VRE was recently published [5]. It showed that approximately 40% of all isolates were positive for at least two genetic elements associated with pathogenicity factors. These data have confirmed the need for the implementation of active surveillance programs that will help in controlling the nosocomial spread of VRE with multiple virulence determinants.

The present study aimed to determine the frequency of and the risk factors for colonization with VRE among Bulgarian critically ill patients who had duration of hospitalization more than 48 h and without infection caused by VRE or/and other microorganisms in the ICU at University Hospital “Dr. G. Stranski”, Pleven from December 2018 to May 2019. During the study period a total of 91 patients fulfilling the aforementioned criteria were screened for intestinal VRE colonization.

Full written consent was obtained from all participants in accordance with the Declaration of Helsinki. The study was approved by the local ethics board of Medical University – Pleven (№ 512/2018). All methods were carried out in accordance with the Medical University – Pleven local guidelines and regulations. Data processing was anonymized and complied with local data protection legislation and with the European Directive on the Privacy of Data (95/46/EC).

In the current case-controlled study all patients with VRE-positive fecal samples and without clinically manifested infection caused by VRE or/and other microorganism were defined as a case group. The control group included patients without detected VRE colonization and infections. The data were collected from patients and their medical records as follows: demographic characteristics (sex and age), clinical information (length of stay in an ICU, reason for admission in an ICU, co-morbidities, usage of invasive devices, etc.) and information about antimicrobials use during the hospital stay.

The statistical analysis of the risk factors for intestinal VRE colonization was performed using SPSS version 27.0 (Armonk, NY: IBM Corp.). First, univariate logistic regression analysis was done for evaluation of the risk factors for VRE acquisition. Additionally, multiple logistic regression was performed for all significant variables in the univariate analysis. Statistical significance was defined as *p* < 0.05.

In our study, VRE colonization was established in 22 of the 91 screened patients (24.2%). One patient was colonized by 2 different *Enterococcus* species. A total of 23 VRE were detected as follows: 9 *Enterococcus casseliflavus* (*vanC2*), 7 *Enterococcus faecium* (*vanA*) and 7 *Enterococcus gallinarum* (*vanC1*). Similar data were presented in an older Brazilian study [6], which identified 5 *E. gallinarun*, 2 *Enterococcus faecalis* and 2 *E. casseliflavus* in screening of 112 patients. Batistao et al. [7] detected 51 *E. casseliflavus*, 26 *E. gallinarum* and 1 VR *E. faecalis* in 333 ICU patients admitted to the University Hospital of Uberlandia, Brazil.

The incidence of VRE colonization varies widely among the ICU patients from different geographic regions. The meta-analysis by Ziakas et al. [8] found that it was highest in the USA (12.3%), followed by Asia (5.3%), Australia (4.4%), and Europe (2.7%). In fecal screening of 256 ICU patients treated in Sao Paulo Hospital, Brazil, colonization with VRE was confirmed in 32.6% of them [9]. Amberpet et al. [10] determined 29% out of 302 ICU patients as carriers of intestinal VRE. In a study on the prevalence of VRE among newborns in ICUs, Duarte et al. [11] found 18.6% VRE intestinal carriage.

Demographic characteristics and clinical data of our colonized patients are presented in Table 1. The medium age of the affected patients was 62.5 ± 14.83 years with predominance of the women. The mean length of hospital stay was 17 days and the main reason for admission to the ICU was the need of postoperative critical cares, which were detected in 36.4% of the patients. Cardiovascular disorders were defined as underling diseases in 72.7% of the colonized individuals.

Using univariate analysis, statistically significant relationship was found between the acquisition of VRE and the following variables: postoperative critical cares (*p* < 0.001), other reasons for admission to the ICU (*p* = 0.003), including traffic accidents, intoxications and suicide injuries, diseases of the cardiovascular system (*p* = 0.009), and the presence of an endotracheal tube (*p* = 0.003) (Table 1). In addition, the postoperative critical cares (*p* = 0.021) and cardiovascular diseases (*p* = 0.018) were confirmed as independent risk factor for VRE colonization by multivariate analysis.

According to an Indian study, the long length of hospital stay (*p* = 0.00), the younger age of the patients (*p* = 0.030), use of ceftriaxone (*p* = 0.25) and vancomycin (*p* = 0.048) were associated with VRE acquisition in ICUs [10]. Hospitalization in the last year (*p* = 0.001), previous use of broad-spectrum antibiotics (*p* = 0.000) or use of two or more broad-spectrum antibiotics in the last year (*p* = 0.009), previous hospitalization in high-risk units (*p* = 0.000), use of immunosuppressive agents (*p* = 0.001) were recently confirmed as risk factors for VRE colonization in a pediatric ICU in Paraguay [11]. The study of Pan et al. [12] showed that the patient’s stay in an ICU was an independent risk factor for colonization with VRE (*p* = 0.03). Also, the authors revealed that the previous use of first-generation cephalosporins was a protective factor against new VRE colonization (*p* = 0.0007). Korean study found that the following variables were significant for prolonged VRE colonization: central venous catheterization and/or endotracheal intubation. In addition, administration of vancomycin after confirmation of VRE colonization makes intestinal carriage of these bacteria 4-fold longer [13].

In conclusion, the population of individuals who need intensive cares consists primarily of critically ill patients requiring intensive treatment and permanent monitoring. Their intestinal screening for VRE colonization will help them from developing subsequent infections with these problematic pathogens. To the best of our knowledge, this is the first study focused on the incidence and risk factors for the occurrence of VRE colonization in ICU patients in Bulgaria. The established prevalence was relatively high (24.2%). It should be noted that this prevalence is referred only to patients without infection caused by VRE or/and other microorganisms. Among the analyzed risk factors, the postoperative critical cares, cardiovascular diseases and the presence of an endotracheal tube contributed to acquisition of intestinal VRE as revealed by the univariate analysis. Multivariate analysis confirmed the first two variables as independent risks factors.

Continuous antimicrobial resistance surveillance, epidemiologic typing of nosocomial VRE isolates as well as screening for VRE intestinal colonization in high-risk patients should be the mainstay of hospital infection control programs.

**Table 1:** Univariate analysis of risk factors for colonization with VRE in the ICU patients studied

Risk factors	Frequency (%) or Mean value ± SD		Univariate analysis	
	VRE Colonized (n = 22)	VRE non-Colonized (n = 69)	OR (95% CI)	*p*-value
Demographics				
Age (years)	62.5 ± 14.83	57.36 ± 16.59	1.670 (0.631–4.418)	0.299
Gender; male: female	7:15	20:49	0.875 (0.310–2.467)	0.800
Mean length of stay (days)	16.86 (3–55)	14 (3-105)	1.560 (0.594–4.094)	0.365
Reason for admission in ICU; n (%)				
Septic shock	2 (9.1)	5 (7.3)	1.28 (0.23–7.11)	0.247
Postoperative critical cares	8 (36.4)	55 (79.7)	0.145 (0.051–0.415)	**< 0.001**
Respiratory failure	3 (13.6)	3 (4.3)	3.474 (0.648–18.632)	0.150
Trauma	2 (9.1)	2 (2.9)	3.350 (0.443–25.319)	0.245
Others*	7 (31.8)	4 (5.8)	7.583 (1.965–29.272)	**0.003**
Underling diseases; n (%)				
Cardiovascular	16 (72.7)	28 (40.6)	3.905 (1.361–11.205)	**0.009**
Respiratory	2 (9.1)	5 (7.2)	1.280 (0.230–7.112)	0.674
Hepatobiliary	1 (4.5)	4 (5.8)	0.774 (0.082–7.311)	0.651
Gastrointestinal	1 (4.5)	3 (4.3)	1.048 (0.103–10.616)	0.969
Genitourinary	1 (4.5)	3 (4.3)	1.048 (0.103–10.616)	0.969
Endocrine	5 (22.7)	14 (20.3)	1.155 (0.363–3.675)	0.772
Malignancies	1 (4.5)	8 (11.6)	0.363 (0.043–3.077)	0.446
Recent abdominal operations	5 (22.7)	21 (30.4)	0.672 (0.219–2.063)	0.486
Use of invasive devices; n (%)				
Central venous catheter	21 (95.5)	57 (82.6)	4.421 (0.541–36.119)	0.176
Drainage tube	16 (72.7)	57 (82.6)	0.561 (0.182–1.731)	0.360
Nasogastric tube	4 (18.2)	17 (24.6)	0.680 (0.202–2.288)	0.531
Urinary catheter	12 (54.5)	42 (60.9)	0.771 (0.293–2.032)	0.599
Endotracheal tube	15 (68.2)	65 (94.2)	0.132 (0.034–0.509)	**0.003**
Antibiotic use during the current hospital stay; n (%)				
Penicillins	1 (4.5)	4 (5.8)	0.774 (0.082–7.311)	0.822
1st generation cephalosporins	1 (4.5)	1 (1.4)	3.238 (0.194–54.036)	0.427
3rd generation cephalosporins	13 (65)	54(76.1)	0.585 (0.201–1.702)	0.322
4rd generation cephalosporins	1 (4.5)	1 (1.4)	3.238 (0.194–54.036)	0.427
Carbapenems	3 (13.6)	4 (5.8)	2.566 (0.528–12.479)	0.353
Aminoglycosides	-	7 (10.1)	0.19 (0.01–3.38)	0.189
Metronidazole	7 (31.8)	31 (44.9)	0.572 (0.207–1.578)	0.278
Fluoroquinolones	1 (4.5)	3 (4.3)	1.048 (0.103–10.616)	0.969
Tigecycline	-	3 (4.3)	0.42 (0.02–8.49)	0.610
Vancomycin	1 (4.5)	3 (4.3)	1.048 (0.103–10.616)	0.969
Antifungal drugs	5 (22.7)	10 (14.5)	1.735 (0.522–5.770)	0.509
Colistin	2 (9.1)	2 (2.9)	3.350 (0.443–25.319)	0.245
Clindamycin	1 (4.5)	2 (2.9)	1.595 (1.138–18.486)	0.569
Linezolid	-	3 (4.3)	0.42 (0.02–8.49)	0.320

**Legend** SD: standard deviation; OR: odds ratio; CI: confidence interval; *Others: Traffic accidents (n = 5); Intoxications (n = 4); Suicide injuries (n = 2)

## Data Availability

All data generated and analyzed during this study are included in this published article.

## List of Abbreviations

CI: Confidence interval
ICU: Intensive care unit
OR: Odds ratio
SD: Standard deviation
VRE: Vancomycin-resistant enterococci
